# Liquid Lipids Act as Polymorphic Modifiers of Tristearin-Based Formulations Produced by Melting Technologies

**DOI:** 10.3390/pharmaceutics13071089

**Published:** 2021-07-16

**Authors:** Serena Bertoni, Nadia Passerini, Beatrice Albertini

**Affiliations:** Department of Pharmacy and BioTechnology, University of Bologna, Via S. Donato 19/2, 40127 Bologna, Italy; serena.bertoni4@unibo.it (S.B.); nadia.passerini@unibo.it (N.P.)

**Keywords:** spray congealing, lipid microparticles, triacylglycerol, drug release, stability, polymorphism

## Abstract

Despite the growing interest in lipid-based formulations, their polymorphism is still a challenge in the pharmaceutical industry. Understanding and controlling the polymorphic behavior of lipids is a key element for achieving the quality and preventing stability issues. This study aims to evaluate the impact of different oral-approved liquid lipids (LL) on the polymorphism, phase transitions and structure of solid lipid-based formulations and explore their influence on drug release. The LL investigated were isopropyl myristate, ethyl oleate, oleic acid, medium chain trigycerides, vitamin E acetate, glyceryl monooleate, lecithin and sorbitane monooleate. Spray-congealing was selected as an example of a melting-based solvent-free manufacturing method to produce microparticles (MPs) of tristearin (Dynasan^®^118). During the production process, tristearin MPs crystallized in the metastable α-form. Stability studied evidenced a slow phase transition to the stable β-polymorph overtime, with the presence of the α-form still detected after 60 days of storage at 25 °C. The addition of 10% *w/w* of LL promoted the transition of tristearin from the α-form to the stable β-form with a kinetic varying from few minutes to days, depending on the specific LL. The combination of various techniques (DSC, X-ray diffraction analysis, Hot-stage polarized light microscopy, SEM) showed that the addition of LL significantly modified the crystal structure of tristearin-based formulations at different length scales. Both the polymorphic form and the LL addition had a strong influence on the release behavior of a model hydrophilic drug (caffeine). Overall, the addition of LL can be considered an interesting approach to control triglyceride crystallization in the β-form. From the industrial viewpoint, this approach might be advantageous as any polymorphic change will be complete before storage, hence enabling the production of stable lipid formulations.

## 1. Introduction

Lipids are applied in a wide range of products within the food, nutraceutical and pharmaceutical fields. In the pharmaceutical industry, solid lipid-based systems, such as microparticles (MPs), pellets and tablets, are commonly used for sustained drug release [1,2] and for achieving taste masking [3]. The incorporation of active ingredients into lipid matrixes can protect labile compounds from different agents (water vapor, oxygen, and moisture) [4,5]. Moreover, lipids are widely employed for the preparation of nanocarriers including liposomes, solid lipid nanoparticles and nanostructured lipid carriers [6,7,8].

The interest and growth in such lipid-based systems can be ascribed to their versatility, low cost, biocompatibility and safety. In fact, most lipid excipients are “generally recognized as safe” (GRAS) [9]. Nevertheless, the complex solid state behavior of lipids is a critical issue. Specifically, triglycerides exhibit a monotropic polymorphism characterized by at least three different crystalline states [10], denoted as α, β′, and β, corresponding to different packing of the hydrocarbon chains and different subcell structures (hexagonal, orthorhombic and triclinic). The various polymorphs differ in their physico-chemical properties including melting point, microstructure, solubility and wettability [9]. As a consequence, polymorphic transitions can profoundly affect the properties of the lipid-based product, including microscopic and macroscopic structural morphology. In the pharmaceutical field, for example, the polymorphic form of triglycerides has been shown to affect exposure to biological processes (e.g., lipolysis, digestion and solubilization) [11] and drug release profiles [12]. In the formulation development of lipid nanoparticles, it has been shown that the metastable polymorphs favor the incorporation of encapsulated molecules, whereas the transition to the stable polymorph results in the expulsion of an encapsulated compound [13]. Even in the food industry, the crystal structure of lipids is extremely important to obtain high-fat products with desired properties. Specifically, the α and β′ forms of triglycerides are more desirable than the β form due to better appearance, texture, and fluidity [14,15]. The β forms may give rise to rough or sandy texture in high-fat products [16].

Solid lipid formulations can be produced with a variety of different methods, most of which are melting-based, such as hot melt extrusion, melt granulation, hot melt coating and spray congealing. These techniques, based on the melting of the lipid followed by re-solidification, can cause the formation of unstable crystalline phases with the tendency to evolve into more stable states during storage. This instability, related to the complex solid state behavior of these materials, is a well-known issue of lipid-containing dosage forms [2,17,18,19]. It is therefore very important to have control over the type of crystalline structure of the lipid-based product during its whole shelf life. Post-process thermal treatments (e.g., curing) is a possible strategy to accelerate the transformation into the stable polymorphic form. However, such treatments increase time and costs of the manufacturing process. It should be also considered that a prolonged exposure to high temperatures might result in a certain drug expulsion, premature drug release and degradation of the loaded active compounds. An alternative strategy is represented by the addition of small amounts of additives acting as “polymorphic modifiers”, i.e., able to modify the crystallization behavior of triglycerides by stabilizing either one or the other polymorphic form.

At present, little attention has been given to the effect of additives on the crystallization of lipid systems during melting-based manufacturing processes and the literature on the role of polymorphic modifiers of lipids is rather scattered. The addition of diacylglycerols and sucrose esters stabilized the metastable forms of triglycerides by retarding the transition to the most stable polymorph [20,21]. Emulsifiers and surfactants were noted to preserve the α-form of solid lipids [22,23]. Specifically, solid surfactants retarded or delayed the polymorphic transitions of tristearin depending on temperature conditions, while some liquid emulsifiers tended to enhance the transformation [24,25]. The effect of amphiphilic additives on the polymorphism of triglycerides has also been noted in the development of lipid systems with dimensions in the nanoscale (e.g., solid lipid nanoparticles, SLN). For example, Helgason and co-workers detected an increase in the number of peaks and a shift to lower temperatures in the melting transition of tripalmitin as the surfactant concentration of Tween 20 in the SLN suspensions increased [26]. Poly(vinyl alcohol) (PVA) stabilized the α-form of tristearin nanoparticles for at least 9 months at refrigerator temperature [27]. The stabilization of the α-polymorph of tristearin nanoparticles achieved by PVA was greater than that of other amphiphilic molecules (sodium glycocholate and sodium glycocholate/phospholipid mixture), although the transition into the stable β-polymorph was not completely prevented [28].

Recently, the addition of polymeric additives (synthetic homopolymers of stearyl methacrylate and oleyl methacrylate) has been shown to impact the crystallization and the crystal habit of tristearin. Depending on the polymer characteristics, the effect resulted either in the stabilization of the metastable (α- or β′) polymorphs or in the modification of the crystals size and morphology [29]. In all the reported studies, however, the purpose of the additives consisted mostly in the stabilization of metastable polymorphs.

To the best of our knowledge, the only paper suggesting the use of additives for stabilizing the stable β-form of solid triglyceride-based formulations was that of Pattarino et al. [30]. In that study, small amounts of medium-chain liquid triglyceride (MCT) added to tristearin-based systems promoted the formation of the stable β-form. As a matter of fact, the effect of MCT in affecting the crystalline structure and stability of tristearin after solidification formed the melt had been previously noted [31]. The stabilization of the β-form could be a successful strategy to produce a stable lipid-formulation without risk of structural changes during storage.

Hence, this study aims to investigate the effect of different oil additives on the crystallization and polymorphism of a solid lipid-based formulation. Glyceryl tristearate (or tristearin) was selected as solid lipid because it is one of the most studied and well-characterized trygliceride with regard to its crystal structures and polymorphic transitions.

As an example of melting-based process, we employed spray congealing, a simple solvent-free process based on the atomization of the molten lipid with obtaining of solid spherical microparticles (MPs). Different oral-approved liquid lipids (LL) were tested for their effect in inducing the crystallization of the most stable polymorphic form (β-form), in order to avoid the slow and uncontrolled polymorphic change overtime. MPs were prepared using commercial tristearin (Dynasan^®^118) alone or with the addition (10% *w/w*) of LL: isopropyl myristate (IM), ethyl oleate, oleic acid, medium chain trigycerides (MCT), vitamin E acetate (Vitamin E), glyceryl monooleate (GMO), soya lecithin (lecithin) and sorbitane monooleate (Span 80).

The effect of the additives on the crystallization, polymorphism and phase transition behavior of tristearin was studied by differential scanning calorimetry (DSC) and hot stage microscopy (HSM). A multi-techniques characterization (DSC, X-ray diffraction analysis, polarized light microscopy (PLM) and scanning electron microscopy (SEM)) was used to investigate the crystal structure of tristearin-based formulations at different length scales. Then, MPs were loaded with a model hydrophilic drug (caffeine) and in vitro release tests were performed in order to explore the influence of polymorphism and LL addition on drug release.

## 2. Materials and Methods

### 2.1. Materials

Tristearin (Dynasan^®^118) was kindly provided by Sasol (Witten, Germany). Caffeine (CAF) and glyceryl monooleate (GMO, 1-Oleoyl-rac-glycerol) were purchased from Sigma-Aldrich (Steinheim, Germany). Tocopheryl acetate (Vitamin E) was purchased from Farmalabor (Milan, Italy). Sorbitane monooleate (Span 80) and isopropyl myristate (IM) were obtained from Fluka (Buchs, Switzerland). Ethyl oleate, oleic acid and liquid soya lecithin were purchased from Carlo Erba Reagents (Milan, Italy). Medium chain trigycerides (MCT, Labrafac™ lipophile WL 1349) supplied from Gattefossè (Milan, Italy). For β-tristearin, Dynasan^®^118 was used as received. α-tristearin was obtained by melting tristearin at 90 °C followed by rapid cooling to room temperature.

### 2.2. Preparation of Microparticles (MPs)

MPs were produced by spray congealing using the wide pneumatic nozzle (WPN) atomizer. Tristearin without or with 10% *w/w* of LL was melted to 5 °C above its melting point. The nozzle temperature and the atomization pressure were set at 5 °C above the carrier melting point and at 1.5 bar, respectively. Upon loading the suspension into the feeding tank, the atomization allowed to the formation of droplets which solidified in the cooling chamber, kept at room temperature. The solid particles were collected and stored at 25 or 4 °C. MPs loaded with caffeine (CAF) were prepared by adding CAF as powder into the melted carriers to form a suspension by magnetic agitation prior to atomization. MPs without LL were produced with 10, 20 and 30% *w/w* of CAF. MPs with LL were produced with 30% *w/w* of CAF.

### 2.3. Particle Size

The size distribution of MPs was evaluated by sieve analysis, using a vibrating shaker (Octagon Digital, Endecotts, London, UK) and seven standard sieves (Scientific Instruments, Milan, Italy) of 50, 100, 150, 200, 250, 355, and 500 μm.

### 2.4. Differential Scanning Calorimetry (DSC)

DSC analysis was performed with a Perkin-Elmer DSC 6 (Perkin Elmer, Beaconsfield, UK). Before measurements, the instrument was calibrated with indium and lead for the temperature, and with indium for the enthalpy. Six to ten milligrams of samples were placed into aluminum pans and analyzed by DSC under a nitrogen flow of 20 mL/min.

Isothermal crystallization study. All scans were started by heating the sample to 90 °C in order to obtain an isotropic melt, and then rapidly cooled to the crystallization temperature (T_cr_) at a rate of 30 °C/min, where the sample was held isothermally for a holding time of 90 min. Tcr in the range 50–62.5 °C were used. Following isothermal crystallization, the sample was heated from the Tcr to 90 °C at 10 °C/min to determine the polymorphic form generated from the preceding isothermal crystallization.

Polymorphic discrimination of tristearin MPs without and with LL. To confirm polymorph identity of the MPs, DSC were carried out, heating the samples from 25 to 90 °C at a scanning rate of 10 °C/min.

### 2.5. Hot Stage Microscopy (HSM) and Hot-Stage Polarized Light Microscopy (HS-PLM)

HSM studies were performed by means of a hot stage apparatus (Mettler-Toledo S.p.A., Novate Milanese, Italy) mounted on Nikon Eclipse E400 optical microscope. Images were taken using a Nikon Digital Net Camera DN100 connected to the microscope which transmitted live images to a computer. MPs of tristearin were directly observed and imaged onto a simple glass slide.

HS-PLM studies were conducted between two crossed polarizers using 10×, 20×, and 40× magnification. For the analysis of the microstructure, MPs of tristearin without and with LL were placed onto a glass slide and melted at 90 °C. The samples were then covered with a coverslip, resulting in a thin film of fat. The slides containing the samples were initially heated to 90 °C and held for 5 min in order to obtain an isotropic melt, cooled to room temperature at a scanning rate of 10 °C/min and imaged after solidification.

### 2.6. Powder X-ray Diffraction (PXRD)

Raw tristearin, MPs without and with LL were studied by X-ray powder diffraction technique using a Philips X’Pert powder diffractometer equipped with a graphite monochromator in the diffracted beam. CuKα radiation was used (40 mA, 40 kV) and the spectra were obtained in the range 5–30° 2θ.

### 2.7. Drug Loading Determination

The drug loading was determined on MPs (main size fraction, corresponding to 250–355 µm) by adding about 10 mg of MPs accurately weighed in 25 mL of water. The sample was heated to 60 °C to melt the carrier and then shaken for 2 h. Finally, the solution was cooled down, filtered, diluted with the same solvent, and the drug content was assayed spectrophotometrically at 273 nm using a Cary-60 UV-Vis spectrophotometer (Agilent Technologies GmbH, Waldbronn, Germany). A calibration curve was prepared with CAF concentrations of 1–20 µg/mL (R^2^ = 0.9996). At least three samples were analyzed for each formulation and results were reported as mean ± s.d.

### 2.8. Release Studies

In vitro release profiles were assessed using the Apparatus 2 (paddle apparatus) of the Eur. Ph. (DT 800 Erweka GmbH, Heusenstamm, Germany) rotating at 100 rpm. The main size fraction, corresponding to 250–355 µm, was used. A suitable amount of MPs containing about 6–8 mg of CAF was added to 500 mL of water kept at 37 °C. At specific time points, 2 mL of the medium were withdrawn using an 8 µm filter to avoid the removal of the MPs and replaced with fresh medium. The amount of drug was determined spectrophotometrically at 273 nm. The results were expressed as CAF released (%) in function of time. Three replicates were analyzed for each formulation.

The release profiles were compared using the similarity factor, f2, according to the equation:

(1) $$f2\;=\;50\;\times \;\log\;\left\{1\;+\;{\left[\;1/n\;\times \;\overset{n}{\underset{t\;=\;1}{\sum }}{\left(\mathrm{Rt}\;-\;\mathrm{Tt}\right)}^{2}\right]}^{-1/2}\times \;100\right\}$$

where n is the sampling number, Rt and Tt are the cumulative percentage dissolved of the reference and the test products at each time point t. Two dissolution profiles are considered similar if the f2 value is between 50 and 100.

### 2.9. Statistical Analysis

All results were expressed as mean ± standard deviation (S.D.). One-way analysis of variance (ANOVA) followed by the Bonferroni post hoc test (GraphPad Prism, version 7.0) was used to analyze the data and the level of significance was set at the probabilities of * *p* < 0.05, ** *p* < 0.01, and *** *p* < 0.001.

## 3. Results

The spray congealing process led to the formation of spherical solid free-flowing particles with size between 50 and 500 µm (Figure S1). The particle size distributions of MPs with LL were similar to MPs without additive, with the most representative particle size fraction corresponding to 250–355 µm, following by the fraction 355–500 µm. Exceptions were MPs with IM, with main fractions in both the range 250–355 µm and 355–500 µm, and MPs with Vit E, showing more than 55% *w/w* of particles >355 µm. The main size fraction (250–355 µm) of MPs was used for further experiments. 

### 3.1. Phase Behavior of Tristearin MPs

#### 3.1.1. Polymorphic Behavior

**Figure 1:**
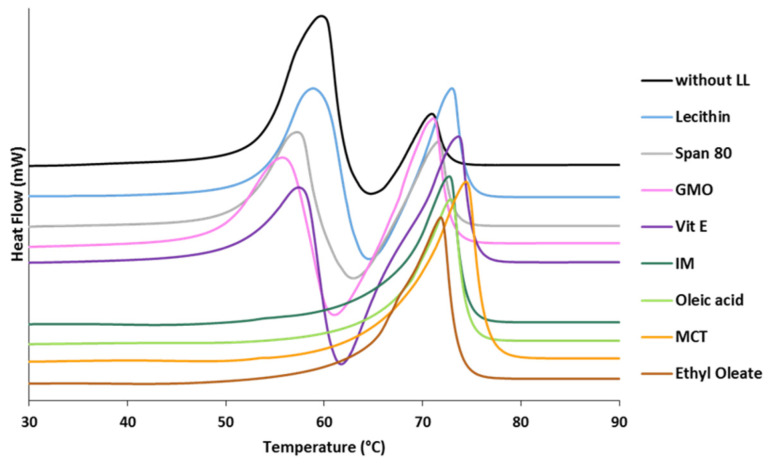
Differential scanning calorimetry (DSC) curve of tristearin MPs without and with LL analyzed immediately after production (heat flow endo up).

The effect of LL additives on the polymorphism of tristearin was studied by DSC by analyzing the MPs immediately after production (within 5 min from MPs collection). Figure 1 shows the DSC profile of tristearin MPs with LL compared to that of pure tristearin. At a scanning rate of 10 °C/min, the curve of tristearin MPs (black curve) exhibited three unresolved events: a melting endotherm at 59.7 °C (with onset at 52.8 °C) followed by an exothermic event at 64.6 °C and a second endothermic peak at 70.9 °C (onset at 68.8 °C). These three events correspond to the melting of the α-form, the crystallization/transformation to the β-form and melting of the β-form, respectively [32].

Differences in the DSC curves were observed for the MPs with LL. Specifically, ethyl oleate, oleic acid, IM and MCT caused the crystallization of the MPs in the β-form during the production process or immediately after. No melting endotherm related to the α-form was detected for the MPs containing these additives. Differently, Vitamin E, GMO, lecithin and Span 80 determined the crystallization of MPs in the α-form, similarly to the MPs of pure tristearin.

**Figure 2:**
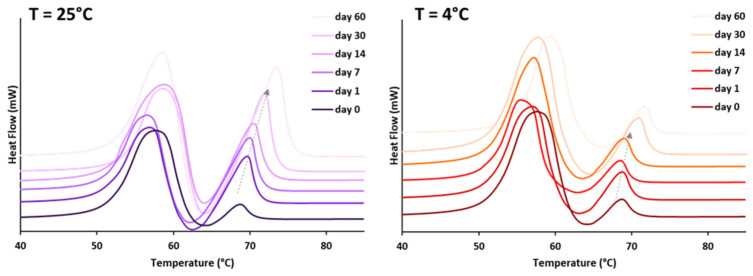
DSC curves of tristearin MPs without LL after storage at room temperature (T = 25 °C) and in the fridge (T = 4 °C) (heat flow endo up).

As a result of processing conditions, solidification in the metastable α-form was observed for tristearin-based formulations without LL or with some additives. The persistence of these unstable polymorphs depends on many factors, e.g., storage conditions. Thus, polymorphic changes of pure tristearin MPs (without LL) were monitored during storage at two different temperatures (Figure 2). Thermograms of samples stored at both temperatures displayed the presence of the α-form up to 60 days of storage. During storage, the progressive development of more ordered β crystals is evidenced by an increased peak enthalpy, especially for MPs stored at 25 °C, and shift to higher temperatures of the β-endotherm. However, the phase transition kinetic was extremely slow and after 60 days of storage the endotherm due to the α-form showed a decrease of only 86% at 25 °C. The storage at low temperature prevented the polymorphic transition, as shown by the negligible increase in the β-endotherm enthalpy (or decrease in the α-endotherm enthalpy).

**Figure 3:**
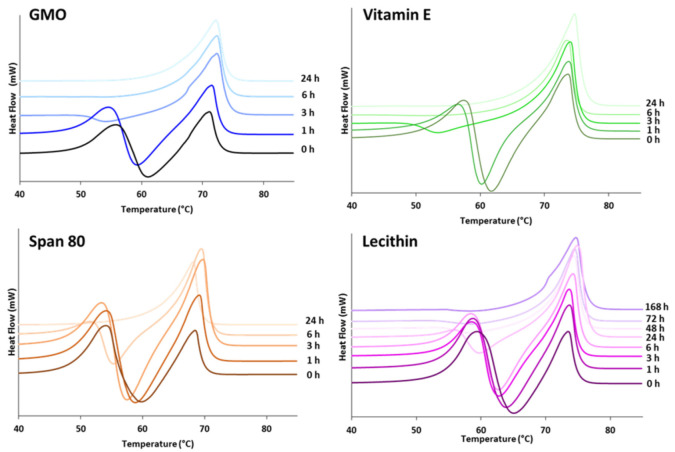
DSC curves of MPs with LL after storage at 25 °C (heat flow endo up).

To better investigate the effect of LL, the polymorphism of MPs with Vitamin E, GMO, lecithin and Span 80 was monitored during storage (Figure 3). The DSC curve of MPs with GMO and Vitamin E showed that the melting endotherm related to the α-form was still present after 1 h from production. However, after 3 h from production, the α-endotherm disappeared and an exothermic event was detected instead. A progressive increase in the peak enthalpy of the β-form was observed, indicating a phase transition to the stable polymorph. After 6 h, only the melting event related to the stable β-form was detected. For MPs with Span 80, the conversion into the β-form was completed after 24 h after production. Finally, when lecithin was used as additive, the conversion in the stable polymorph was completed only after a few days from production.

#### 3.1.2. Crystallization Behavior

First, the crystallization was studied for pure tristearin MPs. Samples of tristearin were melted at 90 °C and crystallized at nonisothermal conditions using a cooling rate of 10 °C/min (Figure S2). The crystallization determined an exothermic peak with an onset at 52 °C and a ΔH_cr_ of −122.06 J/g. Although the nonisothermal conditions closely represents the industrial processing conditions, the crystallization at isothermal conditions allows studying the crystallization kinetic parameters. In the isothermal crystallization experiment, the sample was first heated above its melting temperature to fully melt and was then quickly cooled to the desired temperature. Then, the sample was left crystallized under this temperature and the exothermic event related to the crystallization process was recorded by the instrument. Samples of tristearin were crystallized at isothermal conditions using crystallization temperatures (T_cr_) between 50 and 62.5 °C. This temperature range was chosen as temperatures lower than 50 °C led to early crystallization of tristearin (before reaching the isothermal temperature), whereas temperatures higher than 62.5 °C did not allow tristearin crystallization within the experimental time frame. Original DSC curves of isothermal crystallization are shown in Figure S3.

**Figure 4:**
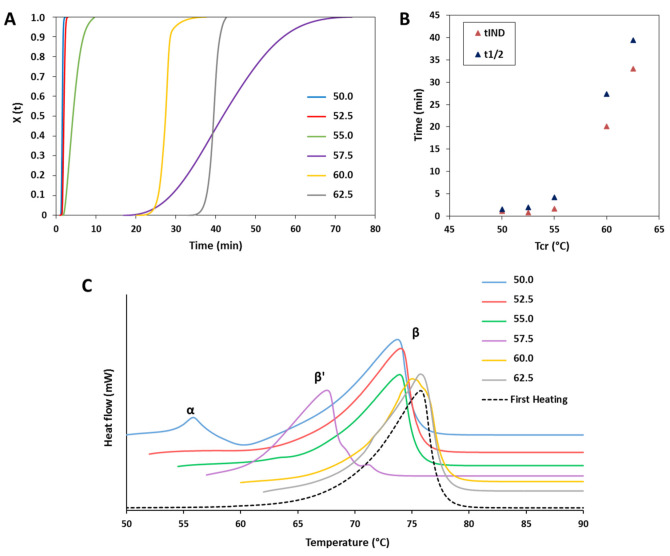
(**A**) Crystallization curves of tristearin at different temperatures (°C) expressed as relative degree of crystallinity (X_(t)_). (**B**) Values of t_IND_ and t_1/2_ calculated for tristearin crystallization at different temperatures. The values for T_cr_ of 57.5 °C are not reported. (**C**) DSC curves of tristearin samples after isothermal crystallization at different Tcr (50.0, 52.5, 55.0, 57.5, 60.0, 62.5) compared with the curve of raw tristearin (heat flow endo up).

Figure 4A shows the isothermal crystallization curves of tristearin expressed as relative degree of crystallinity (X_(t)_) versus time (t), while Figure 4B reports the induction time (t_IND_, the time necessary from the moment the T_cr_ is reached until crystallization starts) and the half-time of crystallization (t_1/2_, the time at which the extent of crystallization is 50%) [33]. After isothermal crystallization, the samples were reheated to 90 °C to determine the polymorphic form (Figure 4C).

Depending on the T_cr_, three different behaviors were observed:

At low T_cr_ (50 and 52.5 °C) where the driving force for crystallization is high, the crystallization process was characterized by very high solidification rates (Figure S2 and Figure 4A). Specifically, the t_IND_ and the t_1/2_ were both extremely low (Figure 4B). Rapid crystallization at low temperatures has been reported to promote crystallization in the α-form [34]. Accordingly, our experimental results showed the presence of α-form upon melting of the crystallized tristearin at 50 °C, but not at 52.5 °C (Figure 4C). However, this can be explained by considering the limit of DSC for small amounts of crystalline solids (lower than 5% *w/w*). Additionally, the presence of the endothermic peak related to the high-melting polymorph (the stable β-form) in samples of α-form is well documented and it is due to the melting of the α polymorph followed by recrystallization in the β-form during DSC scan [31].

Upon increasing the T_cr_ to 55 °C, the solidification rate reduced, together with a longer t_IND_ and t_1/2_. As expected, this trend continues for the crystallization temperatures ≥60 °C. By increasing the T_cr_, the driving force for crystallization is lower and therefore the process takes time to start and develop (higher t_IND_ and t_1/2_), as clearly visible in Figure 4A,B. In particular, the crystallization at 60 and 62.5 °C exhibited characteristically long induction times of 20 and 33 min, respectively, with rapid crystallization following induction time, in accordance with previous studies [34]. At 55, 60 and 62.5 °C, the melt solidified almost completely in the β-form, however, passing from 55 °C to 60–62.5 °C, it can be observed that the endothermic peak of the β melting is moved to slightly higher temperatures (Figure 4C). The appearance of multiple β-melting endotherms may be a consequence of differences in degree of crystal perfection [32]. Accordingly, the melting endotherm of the sample crystallized at the highest temperature (62.5 °C) is identical to that obtained from the first heating of raw tristearin.

At 57.5 °C the crystallization kinetic is visibly different (Figure 4A), with an extremely low crystallization rate as well as very high t_IND_ and t_1/2_ values, not following the trend reported in Figure 4B. Accordingly, the DSC of the crystallized sample showed a melting endotherm at 67.8 °C. This indicates that the melt crystallized into the β′-form.

**Figure 5:**
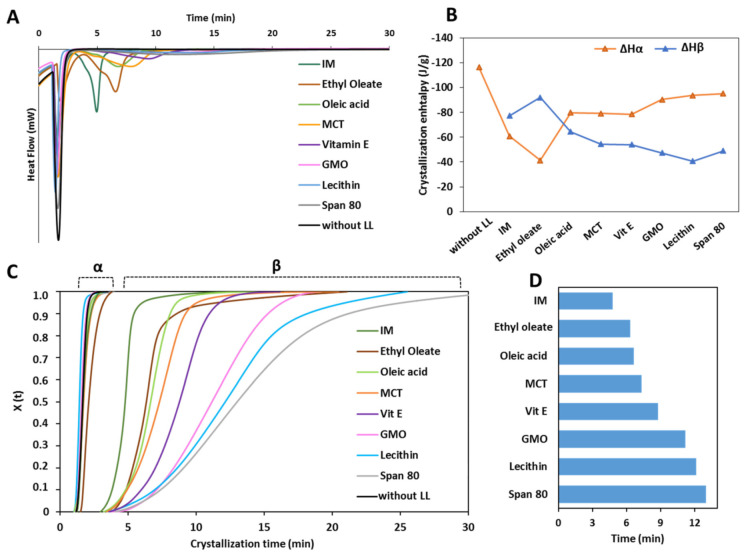
(**A**) DSC curves of MPs without and with LL in the isothermal step at T_cr_ of 50 °C. (**B**) Enthalpy values of the two exothermic peaks (ΔHα and ΔHβ) detected at T_cr_ of 50 °C. (**C**) Crystallization curves of MPs without and with LL at T_cr_ of 50 °C. (**D**) t_1/2_ values calculated from the isothermal crystallization of MPs in the β-form.

Figure 5 shows the results of the isothermal crystallization experiments of MPs containing LL. In the crystallization curves from the melt at 50 °C (Figure 5A) various exothermic events were detected. The first sharp intense peak, observed for all samples, corresponded to the crystallization process of the α-form. This event was extremely rapid and completed within 3.5 min for all samples. For the MPs without LL (black curve), only the crystallization of the α-form was observed. Differently, for MPs with LL, a second exothermic peak was observed after the α-crystallization. This event corresponded to the polymorphic transition from α-form to β-form, as confirmed by a second heating process which showed the presence of the β-polymorphs (data not shown).

The ΔH values of the DSC peaks corresponding to the α-crystallization (ΔHα) and to the β-crystallization (ΔHβ) are reported in Figure 5B. Compared to pure tristearin, LL determined a decrease in the ΔHα to various extents, limited for GMO, lecithin and Span 80, medium for oleic acid, MCT and vit E and marked for IM and ethyl oleate. Specifically, Ethyl oleate caused the most important decrease in ΔHα, which passed from −116.37 to −41.38 J/g. The enthalpy of the second exothermic event followed the opposite trend, i.e., the larger the decrease in the ΔHα caused by LL, the higher the ΔHβ. Notably, the total enthalpy change (ΔHt) of the isotherm crystallization of MPs with LL, was similar for all LL and corresponded to −137.33 ± 4.79 J/g.

The isothermal crystallization curves and the t_1/2_ values calculated from the isothermal crystallization of MPs with LL in the β-form are shown in Figure 5C,D, respectively. After completion of the α-crystallization, the α to β polymorphic transition started within few seconds for all samples with LL. The “S” shape of the crystallization curves of MPs with LL indicated that the β-crystals grow slowly in the beginning, then become faster and slow down at the end of the crystallization process. Interestingly, the crystallization into the β-form followed a different kinetic depending on the type of LL, as indicated by the different slope and shape of the curves. The measured t_1/2_ values of the β-transition of MPs with LL (Figure 5D) evidenced the differences in crystallization rate at which the various LL promoted the α to β transformation. A correlation can be noted between enthalpy change and rate of α→β transition: the higher the value of of ΔHβ, the lower is the time of the β-transition.

### 3.2. Structural Characterization of Tristearin MPs

The structure of MPs with LL at multiple length scale was studied and compared with pure tristearin MPs. MPs were analyzed after 1 week from production (when the polymorphic transition was complete for all LL).

#### 3.2.1. Nanostructural Level

**Figure 6:**
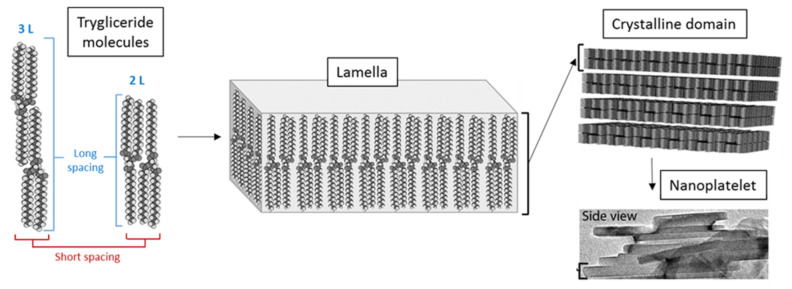
Schematic representation of the special arrangement of triglyceride molecules in pair and subsequently into a lamella. Stacking of several lamellae leads to the formation of a crystalline domain. Adapted with permission from Acevedo et al., Curr. Opin. Colloid Interface Sci.; published by Elsevier, 2011, [38].

At the nanostructural level, the crystallization of tristearin is based on the self-association of tristearin molecules by lateral addition of other molecules, as schematized in Figure 6. These one-molecule thick layers, called lamellae, can be in either a double (2L) or triple (3L) fatty acid chain length structure [35]. Then, the stacking of several lamellae generate a crystalline domain [35]. The distance between the parallel planes of these structures, referred as d-spacing, can be determined from X-ray diffraction. Specifically, the short spacing values in the wide-angle region (15–30° of 2θ) represent the distances between fatty acid chains in the unit cell and are therefore characteristic of the specific polymorph [36]. Differently, the long spacing values in the small-angle region (1–15° of 2θ) correspond to the longitudinal packing and thus are related to the thickness of the lamellae [37]. Specifically, the lamellae thickness of the lipid can be estimated by the d_001_-spacing in the long spacing region. The d_001_-spacing values were calculated from the position at the maximum intensity of the d_003_ reflection detected between 5 and 6° of 2θ in the diffractions pattern, using Bragg’s law, as previously reported [32].

**Figure 7:**
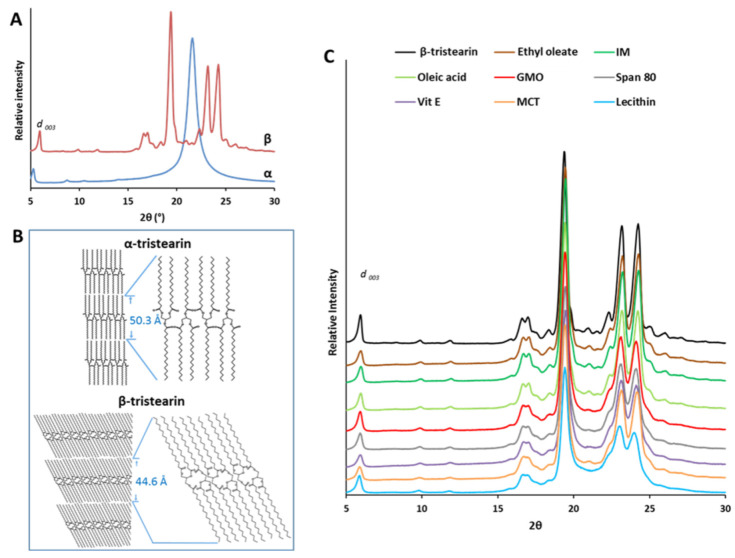
(**A**) XRPD spectra of β-tristearin and α-tristearin MPs. (**B**) Schematic representation of the molecular arrangement of triglyceride molecules in the α and β polymorphs. (**C**) XRPD spectra of MPs with LL compared with pure β-tristearin.

The nanoscale structure of tristearin MPs with and without LL was studied by X-ray diffraction and the results are shown in Figure 7.

Figure 7A shows the XRPD spectra of β-tristearin and α-tristearin. In the wide-angle region, strong reflections were observed at positions 2θ = 19.38°, 23.17° and 24.24° (d-spacing: 4.58, 3.84 and 3.67 Å, respectively). These reflections correspond to the crystal structure of the β-form. Differently, the α-form is characterized by a single strong reflection at 4.13 Å [32,39]. These values are related to the organization of the lateral packing of the alkyl chains of tristearin, corresponding to a hexagonal and triclinic packing of the chains, for α and β-form, respectively [40]. The calculated d_001_-spacing values for β-tristearin and for α-tristearin (Table 1) corresponded to 44.60 Å and 50.27 Å, respectively, similarly to previous results [32,36,41]. These values indicated, for both polymorphs, a lamellar structure with a longitudinal organization of molecules in a double chain-length stacking (2L). The smaller layer thickness (d_001_-spacing) of the β-form compared to that of the α-form depends on the higher density of the crystal packing of the stable polymorph, as schematized in Figure 7B. The scheme evidences that the α and β-form differ from the angle of tilt of the molecules, i.e., the degree of tilting of the hydrocarbon chains plane compared to the end groups axis [42], with increased tilting of the stable form and a tighter molecular packing.

The X-ray diffraction analysis of tristearin MPs containing LL (Figure 7C) confirmed the presence of tristearin in the β-form in all samples. Compared to the β-form of pure tristearin, the diffraction peaks in the short spacing region (15–30° of 2θ) showed minor differences, and a broadening and decrease in intensity was noted for some LL. In particular, this effect was marked for lecithin and MCT, moderate for vitamin E, Span 80 and GMO, and negligible for IM, oleic acid and ethyl oleate. The reflections in the long spacing region (1–15° of 2θ) had similar positions and intensity to the β-form of pure tristearin. In the case of MPs containing ethyl oleate, oleic acid and IM, the d_003_ reflection appears at a higher angle (and hence shorter d_001_-spacing indicating a smaller layer thickness) than pure β-tristearin. Differently, the other LLs led to a slight increase in the d_001_-spacing, indicating a thicker layer and thus a less dense packing of the triglyceride molecules. However, the differences in the positions of the long spacing reflections of MPs with LL compared to that of pure β-tristearin were always smaller than 1 Å (Table 1).

#### 3.2.2. Microstructural Level

The connection between the nanoscale and microscale structure of lipids is not straightforward. Acevedo and Marangoni [38,43,44] have demonstrated that the formation of the lipid crystal network starts with the association of primary crystals with dimensions of about 100 nm called nanoplatelets, formed upon the stacking of several lamellae (Figure 6). The nanoplatelets aggregate via van der Waals’s forces to form the three-dimensional lipid network.

**Figure 8:**
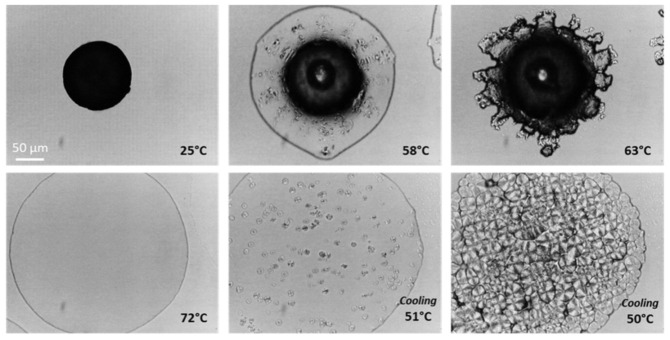
HSM micrographs of tristearin MPs heated from 25 to 90 °C, followed by cooling from 90 °C to 25 °C.

The solid-state transformations of tristearin MPs during heating-cooling processes were observed under a hot-stage microscope (Figure 8). In agreement with the DSC analysis, MPs melted at temperatures of 55–60 °C, but the melting was not complete and the MPs remained partially solid. Then, the melted tristearin re-crystallized at 63 °C, followed by a complete melting at 70–72 °C. In the following cooling step, the melt started to crystallize at 51 °C with the appearance of spherulities, 3-D structures composed of oriented anisotropic lamellar crystallites [17,40]. Spherulities grew separately from the melt as each of them originates from a different nucleus [45]. The microstructure of the fully crystallized tristearin (α-form) consisted of bright well-defined spherulitic structures with diameters of 15–40 µm, in good agreement with previous observations [46].

**Figure 9:**
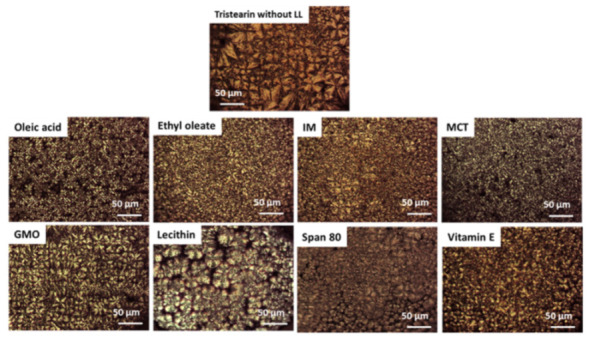
HS-PLM images of crystal structures obtained at 50 °C after crystallization of pure tristearin and tristearin added with LL.

Figure 9 shows the PLM images of MPs with LL cooled from the melt, compared with pure tristearin MPs. Under polarized filters, lipid ordered crystals display birefringence, i.e., they appear bright while materials without ordered structures (e.g., liquid oil) remains dark. Upon crystallization of tristearin MPs without LL, large microstructures (up to 50 µm) were observed. The MPs with LL displayed different crystalline microstructures. Ethyl oleate, oleic acid, IM and MCT determine the formation of a densely packed, homogeneous crystalline pattern of very small, uniform and numerous spherulites. The diameter of the crystals formed was in the range of 1–10 µm. The microstructure of MPs with GMO, lecithin, Span 80 and vitamin E appeared more heterogeneous. The spherulities were larger than those observed for the other LL, though smaller compared to pure tristearin MPs. Specifically, the MPs with lecithin were probably those that closely resemble the microstructure of pure tristearin, displaying the largest spherulities with a size of 20–40 µm. The crystalline network generated in presence of lecithin and Span 80 seems to present imperfections as dark areas among crystal aggregates were observed.

#### 3.2.3. Macrostructural Level

**Figure 10:**
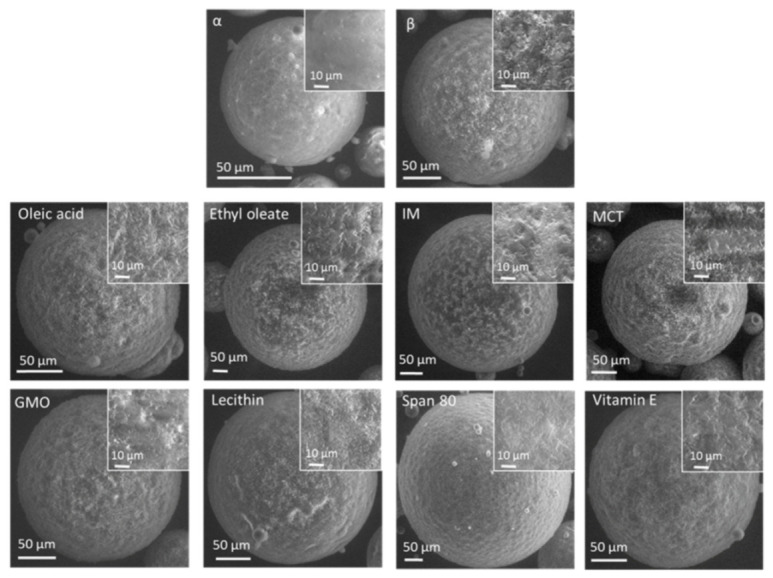
SEM images of particle morphology and magnified surface morphology of MPs without LL (α-tristearin and β-tristearin) and MPs with LL.

The surface morphology of tristearin MPs was studied by scanning electron microscopy (SEM) (Figure 10). All MPs were spherical with comparable size. The surface of freshly-produced MPs (α-form) was smooth, while flake-like structures were observed in the β-tristearin MPs. Similar flake-like structures were observed for MPs containing LL. The definition of these crystalline structures varied for the different LL. Some LL (oleic acid, ethyl oleate, IM, MCT) were characterized by a surface morphology very similar to β-tristearin MPs, whereas other LL (specifically Span 80) determined a smoother surface.

### 3.3. Drug Loading ad Release Profiles of Tristearin MPs

Table 2 shows the composition and drug content of the selected particle size (250–355 µm) of CAF-loaded tristearin-based MPs produced by spray congealing. The drug content values were always close to the theoretical amount.

**Figure 11:**
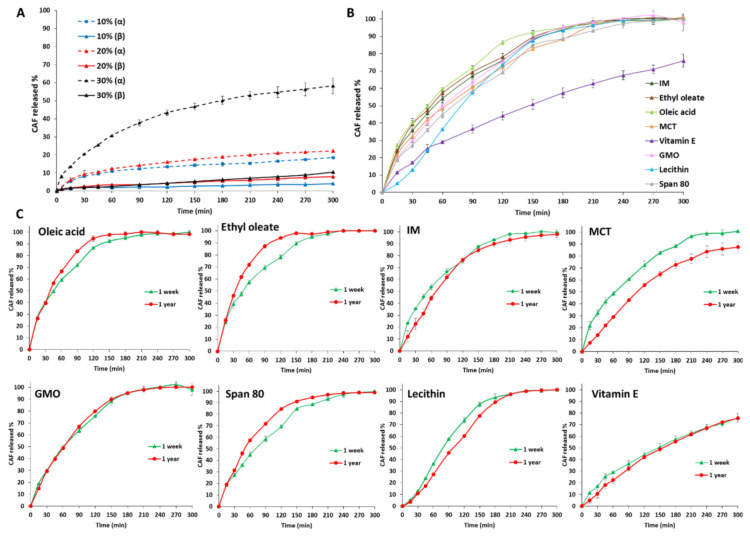
(**A**) Release profiles from MPs of α- tristearin or β-tristearin at different (10, 20 and 30% *w/w*) CAF drug loadings. (**B**) Release profiles from tristearin MPs with LL containing 30% *w/w* of CAF after 7 days from production. (**C**) Comparison of release profiles from tristearin MPs with LL containing 30% *w/w* of CAF before (1 week) and after long-term storage (1 year).

Pure tristearin MPs were produced at three different drug loadings of 10, 20 and 30% *w/w*. The effect of the polymorphic form of tristearin on the drug release was studied by comparing CAF dissolution profiles from pure tristearin MPs in the α-form and in the β-form (Figure 11A). The α-form of MPs was studied immediately after production, while for the β-form, MPs were analyzed after 1 week storage at accelerated conditions (40 °C) upon confirmation of the complete conversion into the stable β-form by DSC. MPs in the β-form led to an extremely slow release of CAF, irrespective of the drug loading (solid lines in Figure 11A). After 5 h, less than 10% CAF was released. MPs in the α-form showed a significantly faster release (dashed lines in Figure 11A). Moreover, for the α-form, the effect of drug loading was significant as MPs with 10, 20 and 30% *w/w* drug content led to 18, 22 and 58% of CAF released by the end of the test, respectively. However, 100% release was not achieved from both polymorphic forms after 5 h.

The effect of LL addition on drug release properties of tristearin MPs is shown in Figure 11B. Likely for structural characterization, MPs with LL were analyzed after 1 week from production in order to allow uniformity regarding polymorphic forms. All the tested LL determined an enhanced release rate. Similar release rates with 100% of drug released already after 3 h were achieved by oleic acid, ethyl oleate, IM, MCT, GMO and Span 80, whereas MPs containing lecithin and vitamin E differ from this profile. The drug release from lecithin-containing MPs showed an S-shaped release profile, with an initial low release rate followed by a gradual increase with time. Finally, a slower release rate without burst effect was observed from MPs with vitamin E as LL, proving to provide the best control of CAF release rate over the tested period of time.

As the goal is to control polymorphic changes overtime, morphology, drug content and release profiles were monitored after long-term storage (1 year) at room temperature. No significant change on particle morphology was observed by SEM analysis (Figure S4) after 1 year storage. Moreover, the drug content of MPs with LL was unchanged (*p* > 0.05), as shown in Figure S5. Additionally, the release profiles (Figure 11C) were mostly unvaried with minor changes. The similarity factor, *f*_2_, were >50 for all LL (IM 70.5; Ethyl oleate 65.0; oleic acid 76.7; MCT 53.1; Vitamin E 81.4; GMO 91.4; lecithin 71.7; Span 80 68.7). The most relevant change in release profile upon storage was observed for MCT, which showed a decrease in release rate compared to day 0, although not significantly different from day 0 (*f*_2_ = 53.1).

## 4. Discussion

Among lipid-based oral drug delivery systems, lipid microparticles (MPs) are versatile drug delivery systems that offer flexibility in both drug release and final dosage form. The wide variety of available matrices and formulation approaches make MPs amenable to a broad range of targeted release kinetics and delivery strategies (taste masking, delayed or controlled release) [47,48,49,50]. MPs can be administered as sachets or incorporated into capsules and tablets or even suspended to obtain a liquid formulation. However, the polymorphism of the majority of lipid excipients, as long-chain triglycerides (e.g., tristearin), is a critical issue for the production of stable formulations. Tristearin exists in three main polymorphic forms: α, β′, and β. This type of polymorphism is monotropic, as one polymorph (the high melting one) is the most stable over the entire temperature range, with all the other polymorphs being unstable. Thus, the polymorphic transformations always occur from a less stable form to a more stable one. Generally, industrial processes involving melting-cooling steps (e.g., spray congealing, hot melt extrusion, melt granulation and melt coating) determined the crystallization in the metastable α-form, which is followed by a slow α→β transition during storage. Polymorphic transitions have been shown to be strongly dependent on processing conditions as well as storage conditions [51]. The stability study of tristearin MPs (Figure 2) showed that the transformation rate was dependent on storage temperature: as storage temperatures increased, the rate of polymorphic transitions from α to β form increased, due to the higher molecular mobility of the triglyceride at elevated temperatures. However, α-form persisted for months at both fridge or ambient temperatures (Figure 2).

DSC analysis revealed that all LL considered in this study, added at 10% *w/w* to tristearin-based formulation, act as polymorphic modifiers. Moreover, their presence influences tristearin structure at different levels. On the basis of their behavior in terms of polymorphic modifications, we can divide the LL in two groups:

- Class I LL (IM, oleic acid, ethyl oleate and MCT) strongly accelerated the polymorphic transition to the stable form, which was completed in few minutes.
- Class II LL (GMO, Vitamin E, span 80 and lecithin) promoted the β phase transition to a lower extent and the time needed to achieve complete polymorphic transition ranged from 6 h to 7 days at room temperature.

The understanding of the underlying mechanisms of the polymorphic and structural changes determined by LL is not straightforward. Overall, all additives tested promoted the polymorphic transition to the stable form. This phenomenon can be associated with a less organized molecular packing of α-crystals in the resolidified melt of tristearin with LL. The presence of liquid additives in the rigid crystalline network provides the molecular mobility required for the rearrangements of tristearin molecules occurring during the α→β transformation. Enhanced polymorphic transitions of bulk triglycerides in the presence of liquid surfactants [24,25] have been explained similarly. The same principle accounts for the storage of tristearin at different temperatures (Figure 2): higher temperatures enhance the extent of mobility of tristearin molecules, accelerating the transformation into the more stable crystal structure.

Crystallization studies provided insights into the mechanisms of LL. Moreover, the crystallization process of a lipid has a strong influence on the final structure, mechanical properties, and functionality of the product. For example, depending on the crystallization conditions (e.g., temperature), different crystal forms of tristearin were obtained by solidification of the melt, as observed in Figure 4. In the isothermal crystallization experiments (Figure 5A), the presence of two consecutive exothermic peaks indicated that LL did not prevent tristearin crystallization in the metastable α-form from the melt. Specifically, as the temperature and kinetics of the α-crystallization of pure tristearin were similar to those of tristearin with LL at the same conditions (Figure 5C), it can be stated that LL had minor influence on the process of tristearin solidification from the melt. This is not surprising as the crystallization of the α-form at low undercooling is kinetically favored. In contrast to the α-form, it has been suggested that for β-form the nucleation is the rate-limiting step of crystallization from the melt (as the β-form has a higher free energy of activation for nucleation) [14,34]. Once β nuclei are formed during the long induction time, the crystals rapidly grow resulting in rapid crystallization rates and thus a steep slope of crystallization curve, as observed during isothermal crystallization of pure tristearin (Figure 4A). Herein, it appeared that LL did not (sufficiently) influence the crystallization kinetics of tristearin as they did not promote the β-crystallization directly from the melt; instead, they accelerate the α→β transformation. Specifically, the enthalpy values of the exothermic peaks related to α-crystallization from the melt and to the α→β transition (Figure 5B) suggested that LL mostly caused polymorphic transformation via solid state, i.e., after completion of α-crystallization. However, the decreased ΔHα and the greater ΔHβ observed for some LL (specifically ethyl oleate and IM, Figure 5B) indicate that a portion of tristearin transformed directly from the melt. For these LL, the stable polymorph is so strongly induced that the crystallization of the β-form started before α-crystallization was completed. The kinetic of the isothermal crystallization of MPs with LL and specifically the t_1/2_ values (Figure 5D) are useful to quantify the extent of LL in inducing α→β transition and thus their “polymorphic modification” potential. The t_1/2_ results are consistent with the polymorphic behavior studied by DSC (Figure 1, Figure 2 and Figure 3) and corroborate the division of LL into the two classes.

An explanation of the LL impact on tristearin phase behavior can be attempted by correlating the observed differences between LL with their physicochemical properties, shown in Table 3.

Overall, IM, ethyl oleate and oleic acid resulted in the LL with the highest “polymorphic modification” potential. Evidently, they have in common specific characteristics allowing them to strongly induce the α→β transition. Among the tested additives, these LL present the lowest molecular weights, the lowest densities and the absence of highly hydrophilic moieties. These properties enable a good “compatibility” between LL and tristearin molecules, thus enabling the incorporation of the additive into tristearin crystalline network. These LL were included in the β-tristearin crystal lattice favoring the compaction and maximizing interactions, leading to a more compact and tight crystalline network (Figure 7C, Table 1). This brings further support for their role in facilitating tristearin molecular mobility. MCT, although classified within Class I LL, differs from the other “fast polymorphic modifiers” as it did not cause a tighter packing of tristearin crystals. However, all class I LL determined a homogeneous crystalline microstructure characterized by regular spherulities and the occurrence of a flake-like surface on the external lipid surface (Figure 9 and Figure 10). The formation of these structures, called “blooming”, is typical of the surfaces of the triglycerides in the β-polymorph [42,52]. Therefore, it appears that the role of IM, ethyl oleate and oleic acid as polymorphic modifiers is related to their ability to fit the crystal lattice of tristearin, determining a tighter packing. Structural similarities between solid triglyceride and additive have been found to facilitate the rearrangement of crystals associated with the polymorphic transformation [53]. In general, it is conceivable that the ability to include an additive in a triglyceride crystalline structure depends on their structural affinity, i.e., the more similar the molecular structures, the higher the chance to fit the foreign molecule in the lipid.

In contrast, Class II LL determined a looser molecular packing of tristearin molecules, as shown by the long spacing values (Table 1). Further, tristearin MPs with class II LL presented a heterogeneous microstructure with larger spherulities and defects. Among the various physicochemical properties, high molecular weight (>400 g/mol) and marked amphiphilic properties seem to induce the polymorphic transition to a lesser extent, as observed for lecithin, Span 80 and vitamin E. Probably, the steric hindrance caused by the hydrophilic group of these additives caused the formation of a loose molecular arrangement and/or defects in the crystalline network, as suggested from the broadening of the diffraction peaks and increased long-spacing values (Figure 7C, Table 1). This observation is in line with the correlation noted by Aronhime et al. [25] between the retardation of the polymorphic transformation of saturated triglycerides and the presence of a hydrophilic moiety of the additive. Among the tested LL, MCT showed an intermediate behavior.

As for the location of the LL within the tristearin crystalline network, two main possibilities can be hypothesized: the additive can either be inserted between the bilayer planes (lamellae) of tristearin crystals or it can be included within the lamellae thickness. In the former case, the structural alteration should determine a change in long spacings of at least 0.5–1 nm [54]. As our results indicated that the variation in long spacings were always smaller than 0.1 nm (Table 1), it is more likely an inclusion of the LL between tristearin molecules due to hydrophobic interactions with the hydrocarbon chains. Clearly, an intermediate situation can also occur, especially if we consider the molecular flexibility of the liquid additives.

From the pharmaceutical point of view, it is fundamental to study whether additives influenced the drug loading capacities and the drug release performances of lipid formulation.

Generally, spray congealing allows high drug loadings with good encapsulation efficiency [55]. There was no loss of CAF during MPs production and LL did not affect encapsulation efficiency, as a CAF loading over 30% was successfully achieved by all formulation produced (Table 2).

In order to understand the factors influencing the release properties of lipid MPs, a consideration of the release mechanism should be carried out. The release of hydrophilic compounds from tristearin systems has been attributed to drug diffusion through the lipid crystal network and/or water diffusion into the system, rather than swelling or erosion [56,57]. Our results indicated that the release of CAF from pure tristearin MPs depended both on drug loading and polymorphism of tristearin. The effect of increased release rate with higher drug amount has been previously observed for MPs [58]. In systems with higher drug loading, the porosity created after the hydrophilic drug started to dissolve probably facilitates further drug release, thus leading to an increased release profile. As for the influence of the polymorphism, the difference in release properties between α and β forms can be associated with the more compact arrangement of the β-crystals compared to the metastable one. Moreover, the “bloomed” surface typical of the β-polymorph impacts the external surface area and the wettability of the MPs, thus influencing drug release. For example, the surface of the β-form of tristearin is more water-repellent compared to the α-form, and the contact angle between water and tristearin increases from 110° to more than 150° passing from the α-form to β-form, respectively [59].

Hydrophilic “pore formers” additives, such as polymers, PEG or Poloxamer have been mostly employed to modify the drug release properties of lipid-based MPs [60]. To the best of our knowledge, no reports evaluate the effect of oil additives on the drug release profiles from triglyceride-based MPs. As shown by the release studies (Figure 11B), the presence of LL strongly enhanced drug release with respect to pure tristearin in the β form. As the release rates from MPs containing amphiphilic LL (e.g., lecithin and Span 80) were not enhanced compared to the others, it is clear that the increase in release does not arise from the surface active properties of LL. Rather, it appeared that other mechanisms are at the basis of drug release from LL-containing tristearin MPs. For example, the release of a hydrophilic drug into the aqueous medium might be favored by the facilitated diffusion through the crystalline network and/or by increased penetration of water into the hydrophobic matrix when LL are present. Similarly, the influence of solid lipid additives (stearic acid, cetyl alcohol, or cetyl esters) was noted to increase the release of paracetamol from spray congealed MPs of paraffin wax [61]. Specifically, LL enhanced CAF release in a similar way with the exception of lecithin and Vit E. The release profile of lecithin-containing MPs exhibited an S-shaped profile with slow release within the first hour (about 35% of drug dissolved) and the 100% of drug release after two hours. Very different was the behavior observed for Vit E-containing MPs, which showed a constant and sustained release of CAF approaching a zero-order release kinetic. A possible explanation could be the marked lipophilicity of vitamin E acetate, with a log *p* (predicted value) around 10 and a calculated lipophilicity value of 14.09 [62]. It is noteworthy that the drug content values and the release profiles showed no significant changes after 1 year of storage (Figure S5 and Figure 11C). Therefore, the strategy of LL addition allowed the production of MPs in the stable tristearin polymorph which avoided changes after storage. On the other hand, the incorporation of LL significantly affected the release profiles of a hydrophilic compound with respect to MPs based only on the pure triglyceride. Clearly, this aspect should be taken into account in view of applications in the oral delivery of active compounds. Nevertheless, the amount of LL and/or the drug loading along with the particle size selection could be adjusted according to the desired release profile and the intended application (e.g., sustained release, taste masking, etc.), thus achieving a balance between polymorphic stabilization and controlled drug release.

## 5. Conclusions

The results demonstrated that the addition of liquid lipids (LL) at a concentration of 10% *w/w* significantly modified the crystallization, phase transitions and microstructure of tristearin-based formulations. Isothermal crystallization studies showed that LL did not affect the crystallization of tristearin in the metastable α-form from the melt, but rather accelerated the α→β polymorphic transition at the solid state. The extent of this effect depended on the type of LL, and specifically the phase transition to the stable form was immediate (few minutes) for LL of Class I, and slower (few hours up to few days) for LL of Class II. The effect of enhanced α→β transition can be explained by considering the role of LL in improving the mobility of the triglyceride molecules. The incorporation of LL altered the lipid structure at the nano-, micro- and macrostructural levels: class I LL determined a tighter molecular packing of tristearin molecules and a densely packed crystalline network, whereas the presence of class II LL caused a loosely molecular packing with microstructural defects. Both the polymorphic form and the LL addition had a strong influence on the release behavior of a model hydrophilic drug (caffeine). Drug content and release profiles of MPs with LL showed no significant changes after 1 year of storage. The effect of lower percentages of LL on the polymorphism and release behavior of tristearin MPs needs further investigation in order to adjust the LL amount according to the target dissolution profile.

Overall, our findings suggest a convenient approach for the stabilization of triglyceride-based systems with potential application in the food, nutraceutical and pharmaceutical fields. This approach allows the production of a stable formulation in terms of solid state and release behavior by promoting the lipid crystallization into the β-form upon solidification after the melting-based manufacturing process.

## Figures and Tables

**Table 1:** X-ray diffraction positions (° of 2 θ) and calculated spacing values (Å) of reflections in the long spacing region of MPs with β-tristearin, α-tristearin and with LL.

	*d _003_*		*d _001_*
	°2θ	Å	Å
Dyn 118 α	5.27	16.75	50.27
Dyn 118 β	5.94	14.86	44.60
Etil oleate	5.96	14.81	44.45
IM	5.98	14.76	44.30
Oleic acid	5.96	14.81	44.45
GMO	5.93	14.98	44.95
Span 80	5.91	14.94	44.83
Vit E	5.91	14.94	44.83
MCT	5.89	14.99	44.98
Lecithin	5.88	15.01	45.06

**Table 2:** Composition and experimental drug amount (% *w/w*) of tristearin-based MPs (250–355 µm size) with and without LL containing CAF.

Formulation	Composition		Experimental CAF Content (%, *w/w* ± SD)
	Amount of Lipid Carrier (%, *w/w*)	Amount of CAF (%, *w/w*)	
Tristearin 10%	90	10	11.53 ± 0.39
Tristearin 20%	80	20	22.57 ± 0.29
Tristearin 30%	70	30	31.35 ± 0.55
IM	70	30	31.98 ± 1.26
Ethyl oleate	70	30	32.66 ± 1.73
Oleic acid	70	30	32.83 ± 0.29
MCT	70	30	33.27 ± 0.28
Vitamin E	70	30	32.99 ± 1.02
GMO	70	30	32.84 ± 0.11
Lecithin	70	30	32.24 ± 0.81
Span 80	70	30	32.78 ± 0.12

**Table 3:** Molecular structure and physicochemical properties of LL.

Liquil Lipids (LL)	Molecular Structure	Molecular Weight (g/mol)	T_m_ (°C)	Density (g/cm^3^) at 25 °C	Hydrophilic Lipophilic Balance (o/w)
Oleic acid	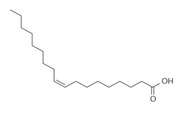	282.5	13–14	0.89	
Ethyl oleate	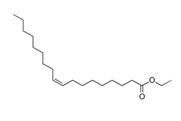	310.5	−32	0.87	-
Span 80	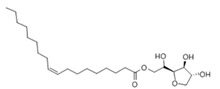	428.6	10–12	0.99	4.6
Glyceryl monoleate (GMO)	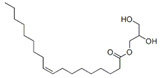	365.5	33–35	0.98	3.4–3.8
Medium chain triglycerides (MCT)	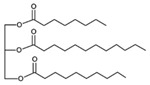	470–640	−5	0.94–0.96	-
Isopropyl myristate (IM)	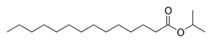	270.5	3	0.85	-
Tocopheryl acetate (Vitamin E)	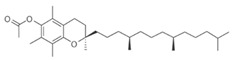	472.7	−28	0.95	-
Soyabean lecithin	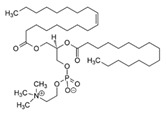	758.1	*	1.05	~7

* not reported in the supplier datasheet.

## Data Availability

This study does not report any data from publicly archived datasets.

## Supplementary Materials

The following are available online at https://www.mdpi.com/article/10.3390/pharmaceutics13071089/s1, Figure S1: Particle size distribution of spray congealed MPs of pure tristearin and with LL addition, Figure S2: DSC curve of tristearin crystallization at nonisothermal conditions, Figure S3: DSC curves of tristearin in the isothermal step at different crystallization temperatures (Tcr), Figure S4: SEM images of particle morphology and magnified surface morphology of MPs without and with LL after 1 year from production, Figure S5: CAF content immediately after production (day 0) and after long-term storage (1 year).
