# Supplementary material for: Liquid Lipids Act as Polymorphic Modifiers of Tristearin-Based Formulations Produced by Melting Technologies
Source: Pharmaceutics. 2021 Jul 16;13(7):1089. doi: 10.3390/pharmaceutics13071089 (PMC8308959; doi:10.3390/pharmaceutics13071089)
Supplement: Supplementary file 1 [file pharmaceutics-13-01089-s001.zip › pharmaceutics-1282537-supplementary.pdf]

pharmaceutics

Supplementary Material: Liquid Lipids Act as Polymorphic Modifiers of Tristearin-Based Formulations Produced by Melting Technologies

Serena Bertoni, Nadia Passerini and Beatrice Albertini

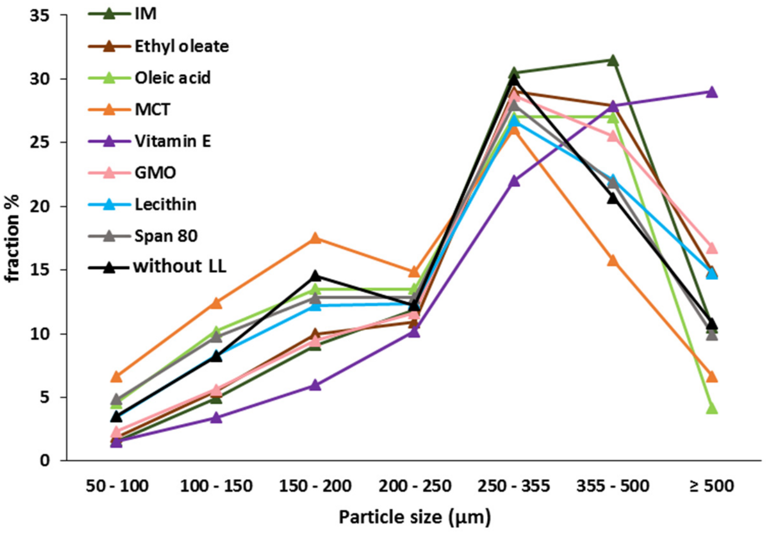

Figure S1. Particle size distribution of spray congealed MPs of pure tristearin and with LL addition.

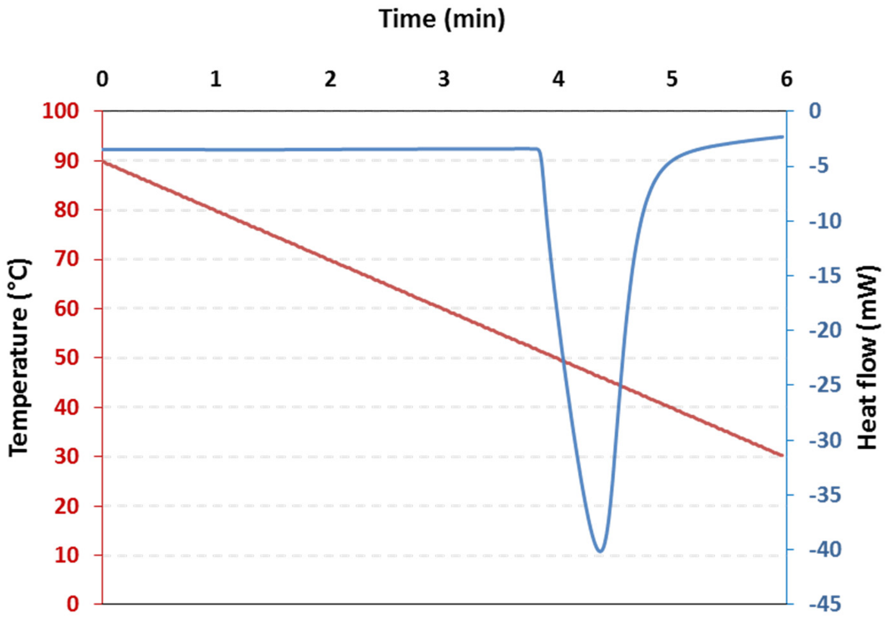

Figure S2. DSC curve of tristearin crystallization at nonisothermal conditions from 90°C to 30°C using a cooling rate of 10°C/min. Both the instrument temperature (red axes) and the measured heat flow (blue axes) are reported.

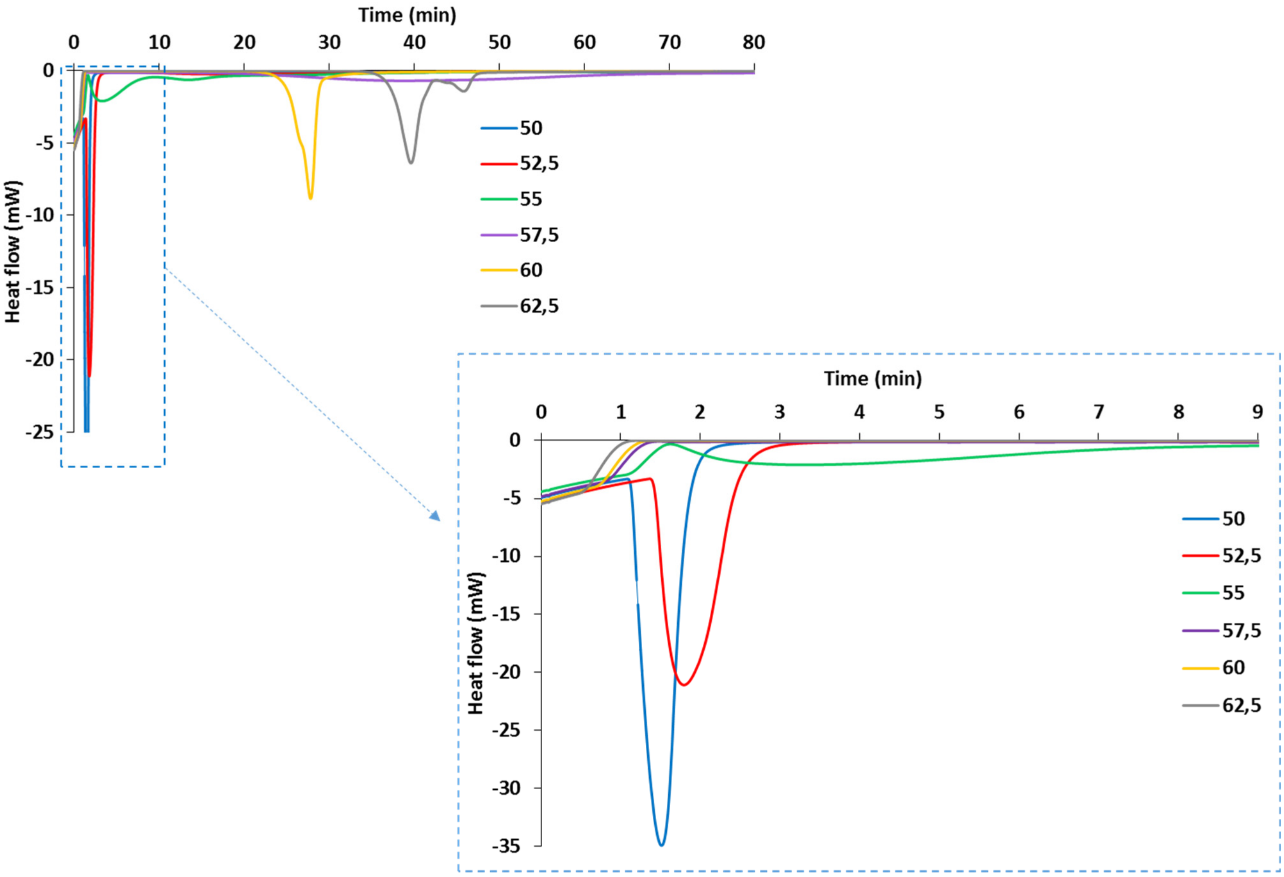

Figure S3. DSC curves of tristearin in the isothermal step at different crystallization temperatures (T_{cr}).

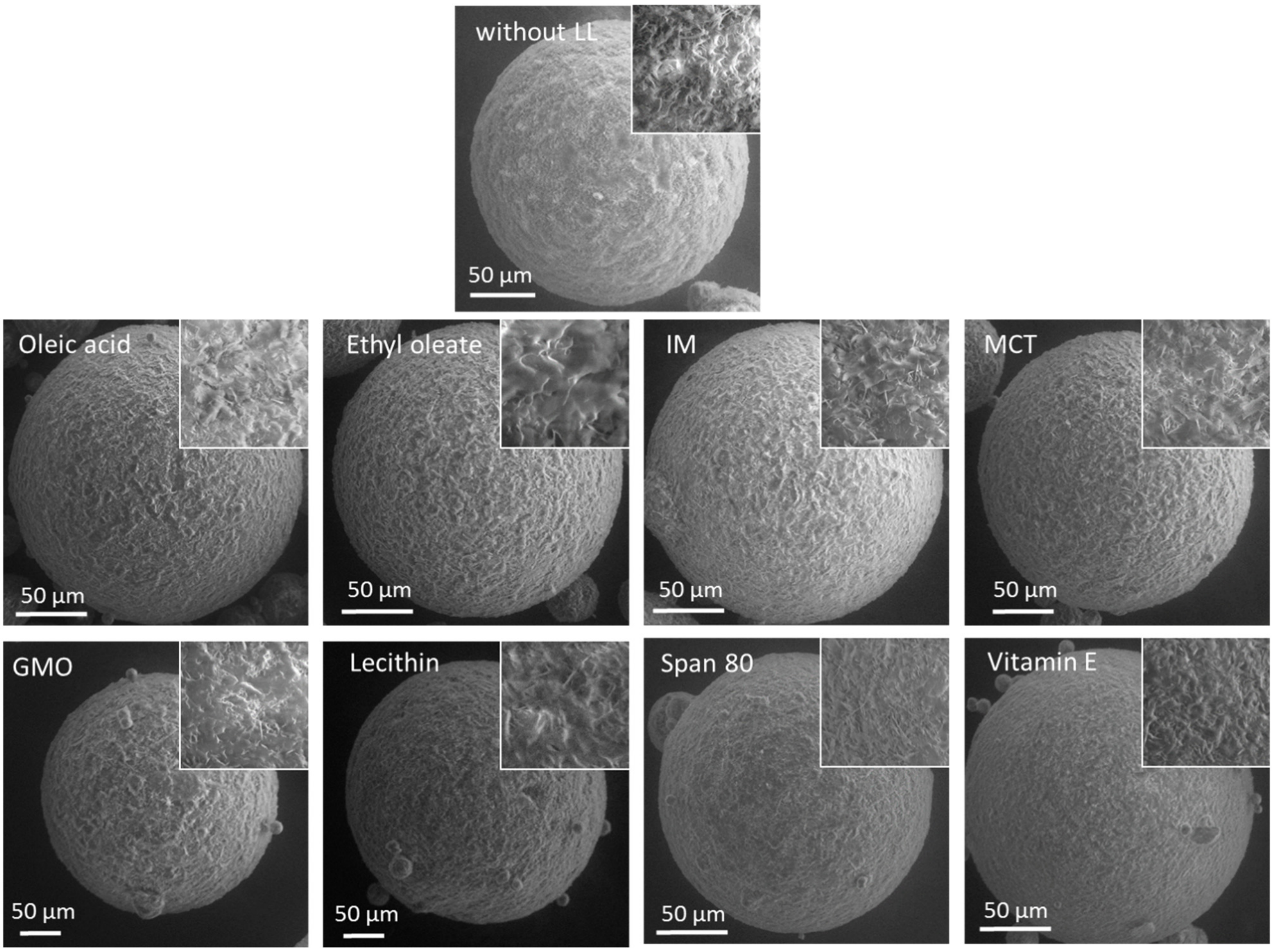

Figure S4. SEM images of particle morphology and magnified surface morphology of MPs without and with LL after 1 year from production.

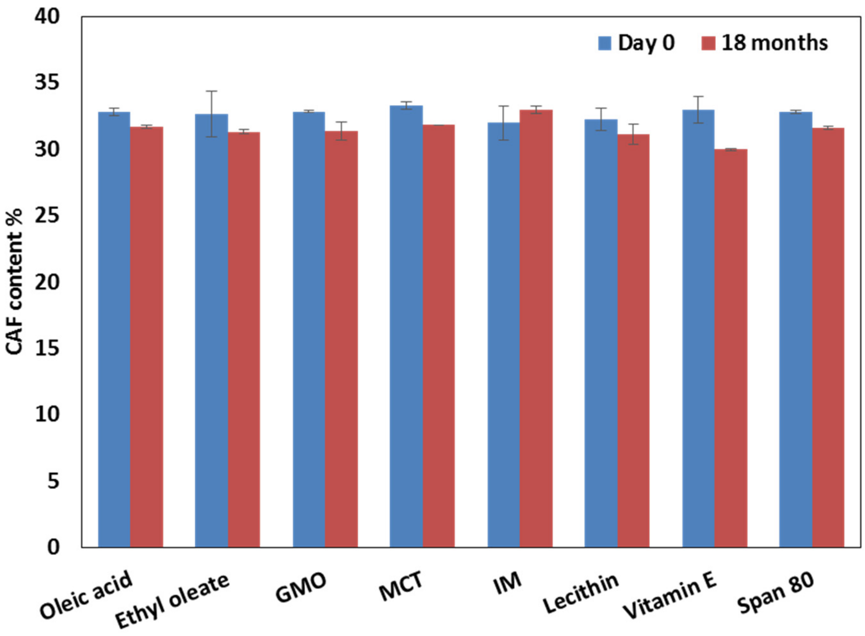

Figure S5. CAF content immediately after production (day 0) and after long-term storage (1 year).
